# Prevention and treatment of suspected pneumonia in Ethiopian children less than five years from household to primary care

**DOI:** 10.1111/apa.15380

**Published:** 2020-06-17

**Authors:** Amare Tariku, Yemisrach B. Okwaraji, Alemayehu Worku, Gashaw Andargie Biks, Lars Åke Persson, Yemane Berhane

**Affiliations:** ^1^ Department of Human Nutrition Institute of Public Health College of Medicine and Health Sciences University of Gondar Gondar Ethiopia; ^2^ Addis Continental Institute of Public Health Addis Ababa Ethiopia; ^3^ Ethiopian Public Health Institute Addis Ababa Ethiopia; ^4^ London School of Hygiene & Tropical Medicine London UK; ^5^ School of Public Health Addis Ababa University Addis Ababa Ethiopia; ^6^ Department of Health System and Policy College of Medicine and Health Sciences Institute of Public Health University of Gondar Gondar Ethiopia

**Keywords:** care utilisation, childhood pneumonia, community case management, Ethiopia, immunisation

## Abstract

**Aim:**

Ethiopia has implemented the integrated community case management to reduce mortality in childhood diseases. We analysed prevention, care seeking and treatment of suspected pneumonia from household to health facility in Ethiopia.

**Methods:**

Analyses were based on a survey in four regions that included modules covering 5714 households, 169 health posts with 276 health extension workers and 155 health centres with 175 staff. Caregivers of children aged 2‐59 months responded to questions on awareness of services and care seeking for suspected pneumonia. Pneumonia‐related knowledge of health workers was assessed.

**Results:**

When a child had suspected pneumonia, 46% (95% CI: 25,68) sought care at health facilities, and 27% (95% CI: 12,51) received antibiotics. Forty‐one per cent had received full immunisation. One‐fifth (21%, 95%: 19,22) of the caregivers were aware of pneumonia treatment. Sixty‐four per cent of the health extension workers correctly mentioned fast or difficult breathing as signs of suspected pneumonia, and 88% suggested antibiotics treatment.

**Conclusion:**

The caregivers' awareness of suspected pneumonia treatment and the utilisation of these services were low. Some of the health extension workers were not knowledgeable about suspected pneumonia. Strengthening primary health care, including immunisation, and enhancing the utilisation of services are critical for further reduction of pneumonia mortality.

**Abbreviations**
CI: confidence interval
WHO: World Health Organization

**Key notes**

- The Ethiopian Ministry of Health invests in improving care seeking for pneumonia and other common childhood illnesses.
- Based on a survey, we show low utilisation of services for children with suspected pneumonia, inadequate coverage of pneumonia‐relevant vaccinations and insufficient knowledge about suspected pneumonia among primary healthcare workers.
- The Ethiopian primary healthcare system needs to improve the prevention and management of suspected pneumonia.

## 1 INTRODUCTION

Pneumonia is the leading cause of childhood mortality in low‐ and middle‐income countries, which contributed to 0.9 million child deaths in 2015.^1^ The World Health Organization recommends the implementation of the integrated community case management programme to reduce mortality in pneumonia and other common childhood illnesses. Many countries, including Ethiopia, have implemented this programme, which has improved the utilisation of services and contributed to the reduction in pneumonia‐related mortality.^2^

Ethiopia achieved a two‐thirds reduction of under‐five mortality during the Millennium Development Goals era.^3^ Still, pneumonia remains the number one killer of children below the age of 5 years in this country.^4^ Following the WHO recommendation, the integrated community case management of childhood illnesses was adopted in 2010 as a national programme for the implementation at the community level through health extension workers. The goal was to enhance the access to treatment for most of the socio‐economically impoverished and hard to reach communities. However, the 2016 Ethiopia Demographic and Health Survey reported that less than a third of caregivers sought health care for their child with suspected pneumonia, and a very few (3%) received antibiotic treatment.^3^ Studies that investigated the background to the low utilisation of integrated community case management of childhood illnesses services have identified demand as well as supply‐side factors. The demand‐side factors included caregivers perceived poor service quality provided at health post,^4^ lack of knowledge on childhood danger signs and available services, and care‐seeking preferences.^5^ The supply‐side factors included the health extension workers' lack of expertise in treating suspected pneumonia cases.^6^ Further, the unavailability of essential drugs and the health extension workers' inadequate supervision and training also contributed to the low utilisation of care.^7^ In response to this, the Ethiopian Ministry of Health, in collaboration with partners, initiated the Optimising Health Extension Programme intervention in 2016, which aimed at improving the utilisation of services provided within the integrated community case management programme.^8^ This intervention was based on a barrier analysis and included three evidence‐based strategies with possible synergies: (a) community engagement activities, (b) capacity building of health extension workers and Women's Development Group leaders, and (c) strengthening the district health services' ownership and accountability of the primary newborn and child health services.

This study aimed to describe the prevention, care seeking and treatment of suspected pneumonia from households to health facilities at the start of the intervention**.**

## 2 METHODS

### 2.1 Study setting, design and population

The basis of Ethiopia's three‐tiered health system is the primary healthcare unit, composed of five health posts and their affiliated health centre. Around 37 000 health extension workers stationed at 18 000 health posts to provide preventive, promotive and essential curative services. This cross‐sectional study was part of the larger baseline survey of the Optimising Health Extension Programme, which was conducted from December 2016 to February 2017 in four regions of Ethiopia, namely Amhara, Tigray and Oromia, and Southern Nations, Nationalities and Peoples regions. These regions represent about 80% of the Ethiopian population. A two‐stage cluster sampling was employed, and a list of enumeration areas was obtained from the Central Statistical Agency based on adjusted figures from the 2007 Ethiopian census and used as a sampling frame. Each enumeration area formed a cluster, which constituted the primary sampling unit. First, two hundred enumeration areas were selected from 52 districts (26 intervention and 26 comparison districts) with probability proportional to size. Second, systematic sampling was applied until a targeted number of 30 households per cluster were selected. Six thousand households were selected from the two hundred enumeration areas.

The sample size was estimated using a double population proportion formula for the evaluation of the effectiveness of the Optimising the Health Extension Programme intervention. The following assumptions were employed: the proportion of children less than five years in the surveyed households was 0.65, and 7% of the children were expected to have suffered from suspected pneumonia during the last 2 weeks.^9^ The design effect was set at 1.3, 5% level of significance, and 80% power was used to detect any differences of 10‐20 percentage points of appropriate care seeking for different common childhood illnesses between baseline and end‐line surveys in intervention and comparison areas. Hence, a total of 6000 households were expected to include 3494 children aged 2‐59 months and 245 cases of suspected pneumonia in children less than five years.

All caregivers of children aged 2‐59 months, who resided in the study districts, were included in the study. For every cluster with 30 households, the corresponding health post and health centre, together with their respective health extension workers and health centre staff serving these clusters, were included in the study.

### 2.2 Data collection

The structured questionnaires used for data collection were developed based on previous literature and major survey instruments. This study was based on one survey that included different questionnaire modules for selected households, health posts that were served by health extension workers, and health centres with their healthcare staff, that is health officers and nurses. The survey questionnaires and data collection tools and procedures were piloted and accustomed to the local context and also translated into the local languages, that is Amharic, Tigrigna and Oromiffa. Data were collected using electronic tablets with logical controls to minimise inaccuracies. There were fifteen data collection teams, and each team comprised of a supervisor and seven data collectors. Data collectors and supervisors held a minimum of first academic degree and were trained for 2 weeks on data collection techniques, procedures, quality assurance and ethical considerations of the study. A supervisor revisited a small sample of households from each cluster to ensure data quality, and the consistency of the original and a repeat interview was compared. Details of the study protocol and tool development are described elsewhere^8^ and have been registered as ISRCTN12040912.

### 2.3 Measurements

In this study, suspected pneumonia was defined as a child aged 2‐59 months who had cough combined with either fast or difficult breathing due to chest problems within the 2 weeks before the survey.^3^ Thus, care seeking was defined as a child with suspected pneumonia for whom advice or treatment was sought from a relevant care provider that included government health facilities or private providers. Moreover, the treatment of suspected pneumonia with antibiotics was estimated as the percentage of children with suspected pneumonia who sought care from an appropriate health provider and received antibiotics.

Child immunisation and vitamin A supplementation are key strategies to prevent pneumonia or mortality from pneumonia.^2^, ^4^ The vaccination status of children aged 12‐23 months was primarily assessed by reviewing immunisation cards. If such cards were absent, the caregivers were asked to report the type of vaccines their children had received. Different probing techniques were used by the data collectors to minimise bias, such as considering the route and timing of the vaccine administration and the potential benefit of the vaccine.

Full immunisation was estimated as the proportion of children aged 12‐23 months who had received BCG vaccination, three doses of pentavalent vaccine, three or more doses of the oral polio vaccine and measles immunisation. Immunisation with the pneumococcal conjugate vaccine was estimated as the proportion of children aged 12‐23 months who had received three doses of this vaccine. Vitamin A supplementation coverage was determined as the proportion of children aged 6‐23 months who had received vitamin A supplement within 6 months before the survey.

The caregivers' awareness of the availability of pneumonia treatment was estimated as the proportion of caregivers who had heard messages regarding pneumonia treatment. The socio‐economic status was represented by a wealth index that was generated by principal component analysis for each household based on ownership of assets. The households were after that categorised into quintiles from the poorest to the least poor.

The pneumonia‐related knowledge of the health extension workers and health centre staff was assessed by asking these health workers to mention signs and management of suspected pneumonia, general danger signs of illness, feeding problems and acute malnutrition. The health workers' knowledge of suspected pneumonia was estimated as the proportion of health extension workers or health centre staff that correctly mentioned cough combined with fast or difficult breathing as signs of suspected pneumonia. The knowledge of signs of severe pneumonia also included stridor or chest in‐drawing.

The health facility‐level treatment of childhood suspected pneumonia was assessed at the health post and health centres by reviewing the registers of sick children 2‐59 months during the last 3 months before the survey. Antibiotic treatment was described as the proportion of children registered to have suspected pneumonia who had received antibiotics.

### 2.4 Data analysis

Descriptive statistics, that is proportions or means along with their corresponding 95% Confidence Interval (CI), were used to summarise the characteristics of the study participants and health facilities. The analyses were performed using STATA version 14 (STATA, Corp).

### 2.5 Ethics approval and consent to participate

This study was approved by the Ethical Review Boards of the Ethiopian Public Health Institute (protocol number SERO‐012‐8‐2016, August 2016), the London School of Hygiene & Tropical Medicine (protocol number 11235, June 2016), and the University of Gondar (V/P/RCS/05/559/2019).

Written informed consent was sought from each participant in the household interview after providing information about the study to each household representative and caregiver. Similarly, written informed consent was also secured from the health extension workers and health centre staff during the health facility data collection.

## 3 RESULTS

### 3.1 Characteristics of caregivers and their children

In this baseline survey, 194 clusters were included. The remaining six clusters were excluded because of social unrest. In these clusters, 2532 caregivers with 3110 children were interviewed. A bit more than half of the caregivers had no formal education. Almost a quarter of the children were aged 2‐11 months (Table 1).

**TABLE 1:** Caregivers' socio‐demographic characteristics, knowledge and care‐seeking preferences in four regions of Ethiopia, December 2016 to February 2017

Variables	Frequency	Percentage (95% Confidence Interval)
Age		
<25	653	26 (24,28)
25‐29	738	29 (27,31)
30‐34	459	18 (17,20)
35‐39	407	16 (15,18)
≥40	275	11 (10,12)
Marital status		
Currently married	2219	93 (92,94)
Unmarried^a^	162	7 (6,8)
Education		
Schooling	1497	59 (55,63)
No schooling	1035	41 (37,45)
Socio‐economic quintiles		
Q_1 (poorest)	524	21 (17,25)
Q_2	492	19 (17,22)
Q_3	513	20 (18,23)
Q_4	516	20 (18,23)
Q_5 (least poor)	487	19 (15,24)
Knowledge of pneumonia treatment		
Knew availability of treatment	702	28 (26,30)
Did not know availability of treatment	1802	72 (70,74)
Preference to HEW^b^ for child pneumonia		
Yes	21	21 (19,22)
No	2008	79 (78,81)
Distance to health facility		
≤30 min	1458	65 (63,67)
>30 min	772	35 (33,37)
Health post visit last 6 mo		
None	1701	67 (65,69)
At least one	831	33 (31,35)
Visited at home by HEW in 6 mo		
Yes	508	20 (19,22)
No	1983	80 (78,81)
Aware of availability of health post		
Yes	2231	89 (88,90)
No	266	11 (10.12)
Aware of availability of WDG^c^		
Yes	186	8 (7,9)
No	2306	92 (91,94)
Participated in community meeting		
Yes	103	4 (3,5)
No	2387	96 (95,97)

^a^ Single, divorced and widowed, and separated and live in union.

^b^ Health extension workers.

^c^ Women's Development Group.

### 3.2 Caregivers' awareness of healthcare services, care‐seeking preferences and utilisation

Less than a third (28%, 95% CI: 26,30) of the caregivers had heard about pneumonia treatment, mainly from health centres or health extension workers at health posts. A majority (89%) of the caregivers were aware of the availability of a health post in their community, and 65% stated that they had <30 minutes' walk to reach the nearest health facility. One‐third (33%) of the caregivers had visited the health post one or more times during the last 6 months. A fifth (21%, 95% CI 19,22) of the mothers preferred to consult the health extension worker if their children had symptoms of suspected pneumonia, while a majority (86%) preferred to seek care at the health centre (Table 1).

**FIGURE 1:**
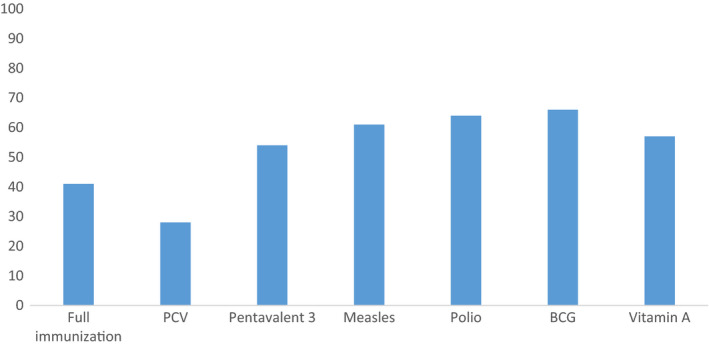
Pneumonia‐specific immunisation and vitamin A supplementation coverage in four regions of Ethiopia, December 2016 to February 2017

When assessed in children aged 12‐23 months, 41% (95% CI: 37,45) were fully immunised and 57% of children aged 6‐24 months had been supplemented with vitamin A. A lower proportion (28%) of children aged 12‐23 months had received the pneumococcal conjugate vaccine (Figure 1).

Overall, 6% (95% CI: 5,7) of the children reportedly had some illness in the last 2 weeks before the survey. Twenty‐two children (0.7%, 95% CI: 0.5,1.1) aged 2‐59 months had suspected pneumonia, of whom ten (46%, 95% CI: 25,68) and six (27%, 95% CI: 20,51) sought care at health facilities and received antibiotics, respectively.

### 3.3 Characteristics of healthcare facilities and providers

This study included 155 health centres and 169 health posts with 175 health centre staff and 276 health extension workers, respectively. Nearly half (48%) of the health extension workers at the health posts had 11 years of education, and 75% of the health centre staff were nurses.

**FIGURE 2:**
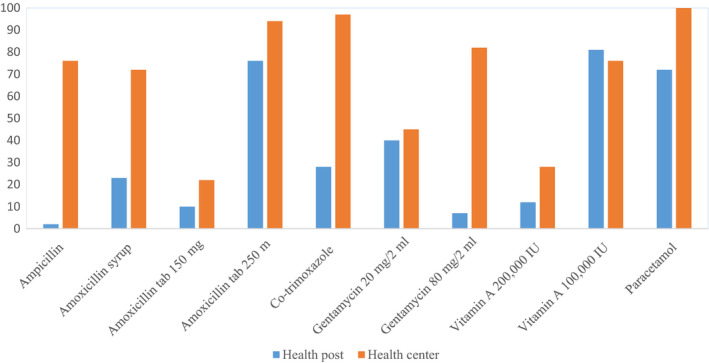
Availability of essential drugs for treatment of pneumonia and vitamin A supplements at health facilities in four regions of Ethiopia, December 2016‐February 2017

Health centres had better availability to antibiotics and vitamin A supplements than the health posts (Figure 2)*.* Approximately one‐quarter (23%) of the health posts had the paediatric concentration of amoxicillin syrup (125 mg/5 mL), while this syrup was found in three‐fourths (72%) of the health centres. Likewise, co‐trimoxazole suspension was more frequently found at the health centres (97%) than health posts (28%)*.*

### 3.4 Healthcare providers' training, supervision and knowledge

A majority (83%) of the health extension workers were trained in the integrated community case management programme, and 78% had received supportive supervision during the 6 months before the survey. In these supervision sessions, 57% had discussed the diagnosis or treatment of pneumonia and 67% about acute malnutrition. Concerning child consultations, over half (53%) of the health extension workers saw children for suspected pneumonia, with an average of ten children in the 3 months before the survey.

Most (89%) of the health centre staff had received training on the integrated management of newborn and child illnesses and two‐thirds (66%) on the diagnosis and management of suspected pneumonia. Most (98%) of the health centre staff were providing curative child health services, with an average of 26 child consultation hours per week. Three‐quarters (74%) of the health centre staff had received supportive supervision*.*

Around two‐thirds (64%) of the health extension workers correctly mentioned both signs of suspected pneumonia, that is cough and fast or difficult breathing. Less than half (46%) recalled either stridor or chest in‐drawing as signs of severe pneumonia. Not all (88%) of the health extension workers recommend antibiotics for suspected child pneumonia, and only 37% recommended a pre‐referral dose of antibiotics for a severely ill child with general danger signs. Few health extension workers and health centre staff were knowledgeable about the signs and management of acute malnutrition and general danger signs of illness (Table 2).

**TABLE 2:** Healthcare providers' knowledge of pneumonia, nutrition and related management in four regions of Ethiopia, December 2016‐February 2017

Knowledge	Health extension workers		Health centre staff	
	N	% (95% CI^a^)	N	% (95% CI)
Signs of pneumonia 2‐59 months sold children				
Cough	209	76 (70,80)	156	89 (84,93)
Difficult or fast breathing	233	84 (80,88)	161	92 (87,95)
Suspected pneumonia (cough and difficult or fast breathing)	178	64(59,70)	143	82 (75,84)
Chest in‐drawing	113	41 (35,47)	131	75 (68,81)
Stridor	64	23 (19,29)	69	39 (32,47)
Either stridor or chest in‐drawing	126	46 (40,52)	142	81 (75,86)
Management of suspected pneumonia				
Give antibiotics	244	88 (84,92)	174	99 (96,100)
Advise on when and how to administer	78	28 (23,34)	65	37 (30,45)
Keep the child warm	33	12 (9,16)	46	26 (20,33)
Advise mother when to return	95	34 (29,40)	76	43 (36,51)
General danger signs				
Unable to drink or breastfed	201	73 (67,78)	143	82 (75,87)
Convulsions	167	61 (55,66)	146	83 (77,88)
Movement or no movement when stimulated	109	40 (34,45)	89	51 (43,58)
Initial management of danger signs				
Refer to higher level	231	84 (79,88)	146	83 (77,88)
Pre‐referral dose and refer to higher level	103	37 (32,43)	93	53 (46,60)
Check if providing oral rehydration solution until reaching facility	47	17 (13,22)	27	15 (11,22)
Signs of newborn feeding problems				
Not well attached to the breast	186	67 (62,73)	99	57 (49,64)
Not suckling effectively	178	65 (59,70)	133	76 (69,82)
Less than eight feeds per 24 h	106	38 (33,44)	70	40 (33,48)
Switching to another breast before one emptied	83	30 (25,36)	39	22 (17,29)
Receive other food or drink, even water	39	14 (11,19)	30	17 (12,24)
Underweight for age	126	46 (40,52)	72	41 (34,49)
Thrush	15	5 (3,9)	11	6 (4,11)
Management of newborns feeding problems				
Advice the mother to breastfeed as often as infant wants in 24 h	135	49 (43,55)	109	62 (55,69)
Teach mother correct positioning and attachment	98	72 (66,77)	126	72 (65,78)
Educate on exclusive breastfeeding	192	70 (64,75)	106	61 (53,68)
Teach the mother to treat thrush at home	45	16 (12,21)	30	17 (12,24)
Follow‐up any feeding problem	74	27 (22,32)	50	29 (22.36)
Follow‐up any thrush in 2 d	20	7 (5,11)	22	13 (8,18)
Follow‐up underweight for age in 14 d	21	8 (5,11)	13	7 (4,12)
Acute malnutrition				
Bilateral pitting oedema	156	57 (51,62)	117	67 (55,74)
Visible severe wasting	179	65 (59,70)	133	76 (69,82)
MUAC^b^ < 11 cm (if 6 mo or older)	249	90 (86,93)	160	91(86,95)
Management for acute malnutrition				
Appetite test if 6 mo or older	129	47 (41,53)	121	69 (62,76)
Give RUTF^c^	216	78 (73,83)	142	81 (75,86)
Advise on when and how to take RUTF	123	45 (39,51)	96	55 (47,62)
Advise when to return	77	28 (23,34)	62	35 (29,43)

^a^ Confidence interval.

^b^ Mid‐upper arm circumference.

^c^ Ready‐to‐use therapeutic food.

### 3.5 Treatment of childhood illnesses at health facilities

A total of 1259 children aged 2‐59 months attended the health posts, while 1490 children attended the health centres in the 3 months before the survey, according to the facility registers. Suspected pneumonia was the leading cause of primary healthcare facility visits with 309 (33%) at the health posts and 442(41%) at the health centres.

Most of the children with suspected pneumonia received antibiotics at the health post and health centres, Table 3. Three‐fourth of children with suspected pneumonia received amoxicillin at the health centres, and fewer were treated at the health posts. The use of co‐trimoxazole for treating pneumonia was more frequent at health posts than at health centres.

**TABLE 3:** Children treated in the last 3 mo at health facilities: characteristics, classification and treatment in four regions of Ethiopia, December 2016‐February 2017

	Health post (1,259)		Health centre (1,490)	
	N	% (95% CI)	N	% (95% CI)
Characteristics				
Age in month				
2‐11	432	35 (32,38)	509	35 (32,37)
12‐23	365	30 (27,32)	407	28 (25,30)
24‐35	221	18 (16,20)	255	17 (16,19)
36‐59	218	18 (16,20)	299	20 (18,23)
Child sex				
Male	682	54 (51,57)	819	55 (52,58)
Female	577	46 (43,49)	671	45 (43,48)
Classification				
Suspected pneumonia	309	33 (30,36)	442	41 (38,43)
Fever	175	19 (16,21)	214	20 (17,22)
Diarrhoea	268	29 (26,31)	227	21 (19,23)
Malnutrition	134	14 (12,17)	23	2 (1,3)
Other	56	6 (5,8)	186	17 (15,19)
Suspected pneumonia				
Mild‐moderate	304	98 (96,99)	436	99 (97,99)
Severe pneumonia	5	1.6 (1,4)	6	1 (0,3)
Treatment				
Suspected pneumonia				
Amoxicillin	197	64 (58,69)	321	73 (69,77)
Co‐trimoxazole	105	34 (29,40)	78	18 (14,22)
Gentamicin	0	0	10	2 (1,4)
No antibiotic	7	2 (1,5)	33	8 (5,10)

## 4 DISCUSSION

We have shown that Ethiopian caregivers' awareness of the availability and utilisation of services for suspected childhood pneumonia and their preference to use the first‐level services provided by health extension workers were low. A majority of the studied children were not fully immunised, that is not protected from the vaccine‐preventable forms of pneumonia. Few of the children with suspected pneumonia received antibiotics. The health extension workers and health centre staff had sub‐optimal knowledge of suspected pneumonia in children. Primary healthcare facilities, especially health posts, frequently lacked essential antibiotics.

We studied both prevention and treatment of suspected pneumonia across household and health facility levels in four of the major Ethiopian regions. Previous studies have either focused on household‐level care utilisation^10^ or facility‐level treatment of suspected pneumonia.^6^ Our study may contribute to a comprehensive understanding of prevention, care utilisation and management across these levels. Recall bias is a potential limitation in reporting illnesses such as suspected pneumonia and immunisations. The illness reporting period was limited to 2 weeks, which is a period considered optimal for common diseases like suspected pneumonia and diarrhoeal diseases. A caregiver's reporting of symptoms that together make suspected pneumonia likely is problematic. Still, we used the questions and definitions that are commonly used in major surveys, such as the Demographic and Health Surveys.^3^ Information on vaccinations was based on the combination of data from immunisation cards and the reporting of the caregiver. Efforts to reduce bias were also made by training of data collectors and supervisors in interview techniques, piloting of the tools and strict supervision of the data collection.

### 4.1 Household‐level knowledge, care‐seeking preference and utilisation

We found that only a quarter of caregivers were aware of pneumonia treatment, which was considerably lower than similar studies in other low‐income countries.^11^ This low awareness could be explained by limited interaction between the health workers and caregivers. The caregiver's awareness of the existence of pneumonia treatment was more limited among those who had never received a home visit by the health extension workers or had never visited the health post. Home visiting by health professionals is considered a cost‐effective strategy to enhance community awareness of childhood illnesses^12^ and improving utilisation of services.^13^ Visiting health facilities also provide opportunities to receive other health information and gain knowledge about child health.^14^

This study showed that only a fifth of the caregivers preferred to contact the health extension workers if their child had suspected pneumonia. Low preference for seeking care from the health extension worker at the health post for childhood illnesses, including pneumonia, was also shown by a previous national study.^15^ The low reported preference for the health extension worker might be related to the caregivers' perception of poor quality of care provided by these health workers coupled to a poor awareness of the availability of pneumonia treatment.^13^ In a previous study, mothers questioned the competence of the health extension workers.^16^ Those who cannot afford treatment at alternative health facilities used the services of the health extension workers at the health post.^5^

We also found a low coverage of child immunisations, particularly of the pneumococcal conjugate vaccination and vitamin A supplementation, which was consistent with the 2016 Ethiopian demographic and health survey.^3^ This coverage was sizably lower than the national target^17^ and suggested that more than half of the children were not protected from the forms of pneumonia, which are preventable by vaccination. Despite this low coverage, Ethiopia has improved the full immunisation coverage during the millennium development goals era from 17% in 2000 to 43% in 2019.^18^

This study showed that less than half of the caregivers sought care when their children suffered from suspected pneumonia. This level of care utilisation is low as compared to reports from other low‐ and middle‐income countries.^19^ The reported low level of care utilisation could be attributed to the caregivers' lack of awareness of available pneumonia treatment^20^ and perceived poor quality of service.^21^

### 4.2 Knowledge of healthcare providers and treatment of pneumonia

Our knowledge assessment of healthcare providers illustrated that two‐thirds of the health extension workers correctly mentioned the signs of suspected pneumonia: nevertheless, less than half recalled signs of severe pneumonia or general danger signs. These findings imply that they lacked the knowledge to classify childhood suspected pneumonia correctly. This problem was further illustrated by another sub‐study of this project, which showed that the health extension workers had great difficulties incorrectly classifying common diseases such as suspected pneumonia.^22^ The reported finding is in line with previously reported inadequate knowledge and management of pneumonia by the health extension workers.^6^ Also, other groups of community health workers in African countries reportedly share similar difficulties.^23^ Such problems could be related to an insufficient capacity building, including training and supportive supervision. The provision of in‐service training and supportive supervision is part of the government's programme.^6^, ^7^ Our analysis showed that nearly a fifth of the health extension workers never received appropriate training. Three‐quarters of these health workers received supportive supervision, which was lower than indicated as an acceptable level, that is >90%.^24^ The health centre staff who supervises the health extension workers should also have the required knowledge and skills.^25^ We found that not all health centre staff were knowledgeable about suspected childhood pneumonia. A previous study also reported a lack of knowledge of the health extension workers' activities among supervisors,^26^ indicating a need of refresher training also to supervisors.

This study reported that only a quarter of children in the household survey with suspected pneumonia received antibiotics, which is lower than similar studies in other low‐income countries.^19^ The facility register review, however, reported that a majority of the children classified to have suspected pneumonia were treated with antibiotics. This difference in reported antibiotic utilisation suggests that caregivers have difficulties in reporting signs and symptoms of suspected pneumonia.^20^

At health facilities, amoxicillin was the most frequently prescribed antibiotic. Still, more than a quarter received co‐trimoxazole. Co‐trimoxazole is less effective in the treatment of pneumonia.^27^ Hence, failing to treat all suspected pneumonia in line with the treatment algorithm and not using the first‐line antibiotics could be related to problems in the supply of essential drugs. Health centres are expected to provide medicines to the health posts, and the scarcity of appropriate antibiotics at the health posts could indicate a lack of these antibiotics or weak linkages between health centres and health posts.

This study is part of an evaluation of a complex intervention that aims at increasing utilisation of primary child health services in Ethiopia.^8^ In another study of the same project, it was revealed that referral practices of sick children were weak from the health post to health centre, and from health centre to hospital.^28^ Children with severe pneumonia were, however, all referred for management at higher level. We showed that the child immunisation coverage was low. Other sub‐studies have revealed that vaccination coverage was equitably distributed from a socio‐economic^29^ and geographic perspective.^30^

## 5 CONCLUSION

The caregivers of children below the age of 5 years had low awareness and utilisation of treatment of suspected pneumonia and pneumonia‐relevant vaccinations. Therefore, demand‐creation strategies to improve community awareness of suspected pneumonia combined with efforts to enhance the quality of care may increase the utilisation of services. Continuous capacity building, supportive supervision, and mentoring of health extension workers and health centre staff are needed, as well as ascertaining the availability of relevant pharmaceutical drugs.

## CONFLICT OF INTEREST

The authors declare that they have no conflict of interest.

## Data Availability

The data for this manuscript were primarily collected by the Ethiopian Public Health Institute and London School of Hygiene & Tropical Medicine. Interested researchers may contact the focal person, Dr Yemisrach B. Okwaraji through email: Yemisrach.Okwaraji@lshtm.ac.uk.

## References

[1] McAllister DA , Liu L , Shi T , et al. Global, regional, and national estimates of pneumonia morbidity and mortality in children younger than 5 years between 2000 and 2015: a systematic analysis. Lancet Global Health. 2019;7(1):e47‐e57. 30497986 10.1016/S2214-109X(18)30408-X PMC6293057

[2] Bhutta ZA , Das JK , Walker N , et al. Interventions to address deaths from childhood pneumonia and diarrhoea equitably: what works and at what cost? Lancet. 2013;381(9875):1417‐1429. 23582723 10.1016/S0140-6736(13)60648-0

[3] Central Statistical Agency [Ethiopia] and ICF International . Ethiopia Demographic and Health Survey 2016. Addis Ababa, Ethiopia and Calverton, MD: Central Statistical Agency and ICF International; 2017.

[4] Deribew A , Tessema GA , Deribe K , et al. Trends, causes, and risk factors of mortality among children under 5 in Ethiopia, 1990–2013: findings from the Global Burden of Disease Study 2013. Population Health Metrics. 2016;14(1):42. 27891065 10.1186/s12963-016-0112-2 PMC5109762

[5] Shaw B , Amouzou A , Miller NP , Tafesse M , Bryce J , Surkan PJ . Access to integrated community case management of childhood illnesses services in rural Ethiopia: a qualitative study of the perspectives and experiences of caregivers. Health Policy Plan. 2015;31(5):656‐666. 26608585 10.1093/heapol/czv115 PMC4857487

[6] Miller NP , Amouzou A , Tafesse M , et al. Integrated community case management of childhood illness in Ethiopia: implementation strength and quality of care. Am J Tropical Med Hygiene. 2014;91(2):424‐434. 10.4269/ajtmh.13-0751 PMC4125273 24799369

[7] Kok MC , Kea AZ , Datiko DG , et al. A qualitative assessment of health extension workers' relationships with the community and health sector in Ethiopia: opportunities for enhancing maternal health performance. Hum Resour Health. 2015;13(1):80. 26423049 10.1186/s12960-015-0077-4 PMC4589131

[8] Berhanu D , Okwaraji YB , Belayneh AB , et al. Protocol for the evaluation of a complex intervention aiming at increased utilisation of primary child health services in Ethiopia: a before and after study in intervention and comparison areas. BMC Health Serv Res. 2020;20(1):339‐412. 32316969 10.1186/s12913-020-05151-3 PMC7171736

[9] Central Statistical Agency [Ethiopia] and ICF International . Ethiopia Demographic and Health Survey 2011. Addis Ababa, Ethiopia and Calverton, MD: Central Statistical Agency and ICF International; 2012.

[10] Astale T , Chenault M . Help‐seeking behavior for children with acute respiratory infection in Ethiopia: results from 2011 Ethiopia Demographic and Health Survey. PLoS One. 2015;10(11):e0142553. 26560469 10.1371/journal.pone.0142553 PMC4641632

[11] Geldsetzer P , Williams TC , Kirolos A , et al. The recognition of and care seeking behaviour for childhood illness in developing countries: a systematic review. PLoS One. 2014;9(4):e93427. 24718483 10.1371/journal.pone.0093427 PMC3981715

[12] Tripathi A , Kabra S , Sachdev H , Lodha R . Home visits by community health workers to improve identification of serious illness and care seeking in newborns and young infants from low‐and middle‐income countries. J Perinatol. 2016;36(S1):S74‐S82. 27109094 10.1038/jp.2016.34 PMC4848742

[13] Yitayal M , Berhane Y , Worku A , Kebede Y . Health extension program factors, frequency of household visits and being model households, improved utilization of basic health services in Ethiopia. BMC Health Serv Res. 2014;14(1):156. 24708661 10.1186/1472-6963-14-156 PMC4234278

[14] Degefa N , Diriba K , Girma T , et al. Knowledge about neonatal danger signs and associated factors among mothers attending immunization clinic at Arba Minch General Hospital, Southern Ethiopia: a cross‐sectional study. BioMed Research International. 2019;2019:1‐8. 10.1155/2019/9180314 PMC6699372 31467919

[15] Mebratie AD , Van de Poel E , Yilma Z , Abebaw D , Alemu G , Bedi AS . Healthcare‐seeking behaviour in rural Ethiopia: evidence from clinical vignettes. BMJ Open. 2014;4(2):e004020. 10.1136/bmjopen-2013-004020 PMC3927812 24525391

[16] Sibamo EL , Berheto TM . Community satisfaction with the urban health extension service in South Ethiopia and associated factors. BMC Health Services Res. 2015;15(1):160. 10.1186/s12913-015-0821-4 PMC4403676 25884574

[17] Federal Ministry of Health, Ethiopia . HSTP Health Sector Transformation Plan 2015/16‐2019/20 (2008‐2012 EFY). Addis Ababa, Ethiopia: Federal Democratic Republic of Ethiopia Ministry of Health 2015.

[18] Central Statistical Agency [Ethiopia] and ICF International . Ethiopia Demographic and Health Survey 2019. Addis Ababa, Ethiopia and Calverton, MD: Central Statistical Agency and ICF International; 2018.

[19] Mosites EM , Matheson AI , Kern E , Manhart LE , Morris SS , Hawes SE . Care‐seeking and appropriate treatment for childhood acute respiratory illness: an analysis of Demographic and Health Survey and Multiple Indicators Cluster Survey datasets for high‐mortality countries. BMC Public Health. 2014;14(1):446. 24884919 10.1186/1471-2458-14-446 PMC4024183

[20] Noordam AC , Sharkey AB , Hinssen P , Dinant G , Cals JW . Association between caregivers' knowledge and care seeking behaviour for children with symptoms of pneumonia in six sub‐Saharan African Countries. BMC Health Serv Res. 2017;17(1):107. 28153011 10.1186/s12913-017-2060-3 PMC5290628

[21] Gage AD , Leslie HH , Bitton A , et al. Does quality influence utilization of primary health care? Evidence from Haiti. Globalization Health. 2018;14(1):59. 29925416 10.1186/s12992-018-0379-0 PMC6011404

[22] Getachew T , Mekonnen S , Yitayal M , ÅkePersson L , Berhanu D . Health Extension Workers' diagnostic accuracy for common childhood illnesses in four regions of Ethiopia: a cross‐sectional study. Acta Paediatr. 2019;108(11):2100‐2106. 31162734 10.1111/apa.14888 PMC7154548

[23] Druetz T , Siekmans K , Goossens S , Ridde V , Haddad S . The community case management of pneumonia in Africa: a review of the evidence. Health Policy Plan. 2013;30(2):253‐266. 24371218 10.1093/heapol/czt104 PMC4325533

[24] World Health Organization . Monitoring and Evaluating Integrated Community Case Management. Geneva, Switzerland: WHO; 2013.

[25] Kok MC , Vallières F , Tulloch O , et al. Does supportive supervision enhance community health worker motivation? A mixed‐methods study in four African countries. Health Policy Plan. 2018;33(9):988‐998. 30247571 10.1093/heapol/czy082 PMC6263021

[26] Datiko DG , Bunte EM , Birrie GB , et al. Community participation and maternal health service utilization: lessons from the health extension programme in rural southern Ethiopia. J Global Health Rep. 2019;3:e2019027.

[27] World Health Organization . Revised WHO Classification and Treatment of Childhood Pneumonia at Health Facilities. Geneva, Switzerland: WHO; 2014. 25535631

[28] Beyene H , Hailu D , Tadele H , Persson LÅ , Berhanu D . Insufficient referral practices of sick children in Ethiopia shown in a cross‐sectional survey. Acta Paediatr. 2020;40(2):187. 10.1111/apa.15200 PMC7496527 31999877

[29] Wuneh AD , Medhanyie AA , Bezabih AM , Persson LÅ , Schellenberg J , Okwaraji YB . Wealth‐based equity in maternal, neonatal, and child health services utilization: a cross‐sectional study from Ethiopia. Int J Equity Health. 2019;18(1):1‐9. 31870447 10.1186/s12939-019-1111-2 PMC6929360

[30] Defar A , Okwaraji YB , Tigabu Z , Persson LÅ , Alemu K . Geographic differences in maternal and child health care utilization in four Ethiopian regions; a cross‐sectional study. Int J Equity Health. 2019;18(1):173. 31718658 10.1186/s12939-019-1079-y PMC6852737

